# Atrial Fibrillation and HFrEF: Cause, Consequence, or Both?

**DOI:** 10.3390/biomedicines14091922

**Published:** 2026-08-27

**Authors:** Mihai Grigore, Andreea-Maria Grigore, Ruxandra-Elena Martin-Graur, Camelia Nicolae, Ana-Maria Balahura, Verde Ioana, Uscoiu Gabriela, Adriana-Mihaela Ilieșiu

**Affiliations:** 1 Cardio-Thoracic Department, Carol Davila University of Medicine and Pharmacy, 021021 Bucharest, Romania; mihai.grigore@umfcd.ro (M.G.); camelia.nicolae@umfcd.ro (C.N.); ana-maria.balahura@umfcd.ro (A.-M.B.); adriana.iliesiu@umfcd.ro (A.-M.I.); 2 Internal Medicine and Cardiology Department, “Prof. Th. Burghele” Clinical Hospital, 050653 Bucharest, Romania; 3 Cardiology Department, Colentina Clinical Hospital, 020125 Bucharest, Romania; ruxandra-elena.martin@drd.umfcd.ro

**Keywords:** heart failure with reduced ejection fraction, atrial fibrillation, comorbidity, prognosis

## Abstract

**Background/Objectives:** Atrial fibrillation (AF) and heart failure with reduced ejection fraction (HFrEF) frequently coexist and are linked by shared risk factors and pathophysiological mechanisms. Their association is consistently related to worse clinical outcomes. This narrative review summarizes current evidence regarding the epidemiology, pathophysiology, management strategies, and prognosis of patients with AF and HFrEF. **Methods:** This is a narrative review based on published randomized trials, registries, and observational studies evaluating the interaction between AF and HFrEF, with a focus on therapeutic strategies including rate and rhythm control, catheter ablation, stroke prevention, and guideline-directed medical therapy. **Results:** AF and HFrEF have a bidirectional relationship, with each condition promoting the development and progression of the other. AF contributes to hemodynamic impairment and adverse remodeling, while HFrEF promotes atrial structural and electrical changes. Management remains complex. Rate control is commonly achieved with beta-blockers and digoxin, while rhythm control is limited by the safety profile of antiarrhythmic drugs. Catheter ablation has shown improvements in left ventricular function and, in selected patients, reductions in hospitalizations and mortality, although results are not consistent across all trials. Guideline-directed medical therapy improves outcomes in HFrEF, but its interaction with AF varies across drug classes. Stroke risk is significantly increased, requiring appropriate anticoagulation. Patients with AF and HFrEF have an increased risk of mortality, hospitalization, and adverse clinical events compared with those with either condition alone, although the extent to which these outcomes are attributable to AF itself, HF severity, or the associated comorbidity burden remains uncertain. **Conclusions:** The coexistence of AF and HFrEF identifies a high-risk population with complex management and unfavorable outcomes. While advances in rhythm control and heart failure therapies have improved selected outcomes, optimal treatment strategies and their impact on long-term prognosis remain incompletely defined.

## 1. Introduction and Epidemiology

Atrial fibrillation (AF) is the most common sustained cardiac arrhythmia, currently affecting approximately 10.5 million individuals in the United States and 11.7 million people in the European Union [1,2]. In parallel, heart failure (HF) represents a major and growing global health burden, with over 64 million individuals affected worldwide, of whom nearly 50% present with heart failure with reduced ejection fraction (HFrEF), defined by a left ventricular ejection fraction (LVEF) ≤ 40% [3,4]. Projections suggest a further rise in prevalence, with AF in the United States expected to increase from approximately 5 million in 2010 to 12 million by 2030, while HF is estimated to affect more than 8 million adults by the same time point [5].

AF and HFrEF frequently coexist and are linked by shared risk factors—including advanced age, structural heart disease, hypertension, obesity, and diabetes—as well as overlapping electrophysiological, hemodynamic, autonomic, and neurohormonal disturbances that promote adverse cardiac remodeling [6,7,8]. Their coexistence is associated with a substantially increased risk of adverse outcomes, including all-cause and cardiovascular mortality [9,10], stroke, and progressive HF worsening [11]. Contemporary registry and cohort data indicate that AF is present in approximately one-third of patients with HFrEF, with prevalence rising steeply with HF severity, from less than 5% in New York Heart Association (NYHA) functional class I to up to 50% in NYHA class IV However, this association should not be interpreted as evidence of a direct causal relationship between AF and HF severity, as it may partly reflect the greater burden of advanced age, left atrial remodeling, elevated filling pressures, neurohormonal activation, and comorbidities in patients with more advanced HF [11,12].

Beyond simple coexistence, epidemiological data support a bidirectional and asymmetric temporal relationship between AF and HF. The lifetime risk of developing AF is estimated at 22–36%, depending on sociodemographic characteristics [13]. Among patients hospitalized for HF, AF is identified in up to 41% of cases and is associated with increased healthcare costs and mortality [14]. Longitudinal analyses from the Framingham Heart Study, including patients with HF irrespective of ejection fraction, demonstrate that AF precedes the development of HF three times more frequently than AF develops after an HF diagnosis, and that individuals with AF have a 4.6-fold increased relative risk of subsequently developing HF [12,15].

This narrative review aims to provide a clinically oriented synthesis of the complex relationship between AF and HFrEF, integrating evidence from observational studies, randomized clinical trials, meta-analyses, and current clinical guidelines. Particular emphasis is placed on the heterogeneity of the AF–HFrEF interaction, its therapeutic implications, and areas of persistent uncertainty. Given the narrative nature of this review, no formal systematic search strategy or predefined inclusion and exclusion criteria were applied.

## 2. Pathophysiological Interplay Between Atrial Fibrillation and Heart Failure with Reduced Ejection Fraction

### 2.1. Atrial Fibrillation Leading to Heart Failure

The frequent coexistence of atrial fibrillation (AF) and heart failure (HF) reflects both shared risk factors and the ability of each condition to promote the development of the other [16]. Aging-related mechanisms, including oxidative stress and progressive myocardial injury affecting both atrial and ventricular myocardium, contribute to structural remodeling that predisposes to either AF or HF [17,18].

AF may precipitate or worsen HF through several hemodynamic and neurohormonal mechanisms, including tachycardia-induced cardiomyopathy, irregular ventricular rhythm, loss of atrial contraction, functional mitral regurgitation, and activation of the renin–angiotensin–aldosterone system (RAAS) [19,20,21,22]. Persistent tachycardia and rapid ventricular rates can impair left ventricular (LV) function by increasing ventricular pressures, altering calcium handling, and shortening diastolic filling time, ultimately reducing cardiac output and promoting adverse myocardial remodeling [19]. In addition, the irregular ventricular rhythm characteristic of AF may further decrease cardiac output, while the loss of coordinated atrial systole—normally contributing up to 25% of ventricular filling—impairs ventricular preload and overall cardiac performance [20,21]. Structural atrial remodeling and dilation may also lead to functional mitral regurgitation, increasing ventricular filling pressures and contributing to progressive ventricular dysfunction [22,23].

In some patients, sustained AF may lead to AF-mediated cardiomyopathy, a form of arrhythmia-induced cardiomyopathy characterized by reversible LV systolic dysfunction that improves after restoration of sinus rhythm [24,25]. This condition may develop within weeks to months after AF onset, particularly in the presence of persistent tachycardia, although additional contributors such as irregular ventricular rhythm, impaired myocardial flow reserve, and loss of atrial contraction also play important roles [4,24]. Experimental studies further suggest that AF may impair ventricular excitation–contraction coupling through oxidative stress and abnormalities in calcium handling, even at normal heart rates [24]. Restoration of sinus rhythm, particularly through catheter ablation, has been associated with significant improvement in LV function, highlighting the direct contribution of AF to ventricular dysfunction [25,26]. AF may also reduce cardiac output by approximately 20% and promote functional atrioventricular valve regurgitation, which further contributes to atrial dilation and progressive ventricular remodeling [27,28].

### 2.2. Heart Failure Leading to Atrial Fibrillation

Conversely, HF promotes the development of AF through increased cardiac filling pressures and atrial structural remodeling. Both systolic and diastolic LV dysfunction lead to elevated ventricular and atrial pressures, resulting in atrial stretch and increased wall stress [29]. These mechanical changes activate neurohormonal, inflammatory, and pro-fibrotic pathways that contribute to atrial myopathy and electrical instability [10,23].

Volume overload and atrial dilation further reduce atrial conduction velocity and refractory periods while increasing heterogeneity of atrial repolarization, thereby facilitating re-entry and AF initiation [30,31]. Structural alterations such as atrial fibrosis and conduction disturbances further disrupt electrical continuity within atrial myocardium and promote the maintenance of AF [32,33,34,35].

At the cellular level, abnormalities in calcium handling also contribute to atrial arrhythmogenesis. Increased Ca^2+^/calmodulin-dependent kinase II activity can induce hyperphosphorylation of ryanodine receptors and abnormal sarcoplasmic reticulum calcium leak, generating delayed afterdepolarizations and triggered activity [36,37,38]. Similar alterations in calcium cycling and ion channel regulation have been described in experimental models of HFrEF, contributing to atrial electrical instability [39,40]. In addition, autonomic imbalance and neurohormonal activation associated with HF may further enhance AF susceptibility and arrhythmia maintenance [4].

### 2.3. Beyond Bidirectionality: Heterogeneity of the AF–HFrEF Interaction

Beyond the well-established bidirectional relationship between AF and HFrEF, their coexistence may evolve into a more complex pathophysiological phenotype as the disease substrate progresses. In this setting, the dominant driver may become increasingly difficult to identify, as hemodynamic impairment, atrial and ventricular structural remodeling, fibrosis, electrical abnormalities, and neurohormonal and autonomic activation progressively converge into a self-perpetuating substrate. Importantly, correction of the initial trigger does not necessarily result in complete reversal of this substrate. Although LV systolic function may recover following restoration of sinus rhythm, residual ventricular remodeling, atrial fibrosis, and conduction abnormalities may persist. Experimental evidence similarly suggests that reversal of HF-related ionic remodeling after hemodynamic recovery does not necessarily abolish the arrhythmogenic substrate, further supporting the role of persistent structural remodeling in AF maintenance [4].

**Figure 1:**
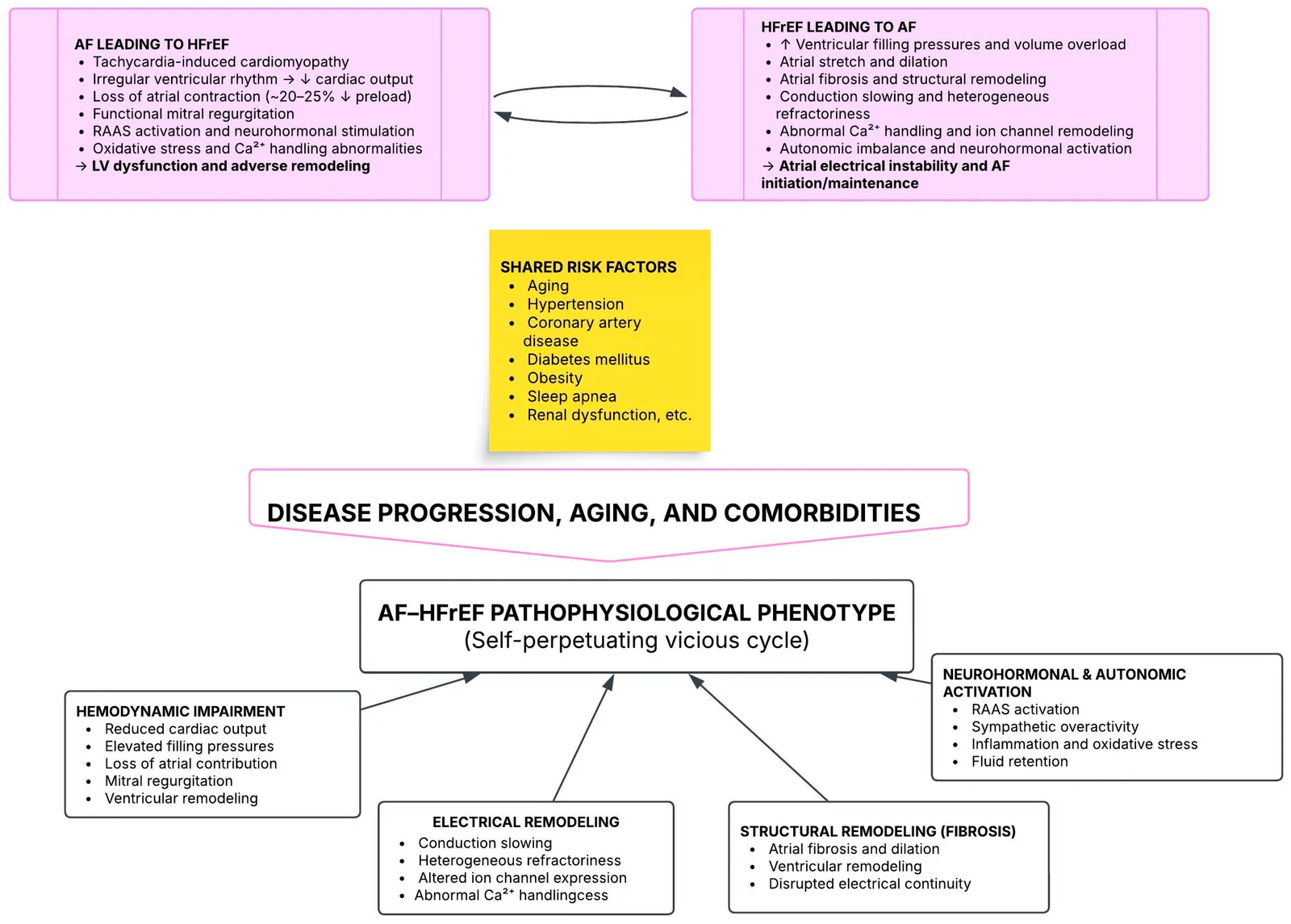
From Bidirectional Interaction to a Distinct AF–HFrEF Pathophysiological Phenotype.

Thus, particularly in patients with advanced cardiovascular disease and a substantial comorbidity burden, AF and HFrEF may become difficult to classify simply as cause or consequence. Their coexistence may instead reflect a complex AF–HFrEF pathophysiological phenotype in which hemodynamic dysfunction, electrical and structural remodeling, fibrosis, and neurohormonal activation interact within a self-perpetuating vicious cycle, progressively sustaining both atrial arrhythmogenesis and ventricular dysfunction. Whether this phenotype can be reliably identified, which mechanisms predominate in individual patients, and whether its recognition may predict therapeutic response, particularly to rhythm-control strategies, remain important unresolved questions (Figure 1) [4].

## 3. Management of Atrial Fibrillation in HFrEF: Rate Control, Rhythm Control, and Stroke Prevention

### 3.1. Rate and Rhythm Control

The choice between rate and rhythm control in AF depends on clinical factors such as age, comorbidities, AF type and duration, and especially symptom burden. Rhythm control is generally favored in patients who remain symptomatic or experience worsening HF symptoms, reduced exercise capacity, or left ventricular dysfunction during AF [41].

#### 3.1.1. Rate Control

Rate control aiming for <110 beats/min appears comparable to stricter targets (<80 beats/min), as no additional benefit of strict control has been demonstrated for mortality, morbidity, symptoms, or quality of life [42].

In clinical practice, ventricular rate control is primarily achieved using pharmacological agents, including:

##### Beta-Blockers

Beta-blockers are a central component of guideline-directed therapy in patients with HFrEF and are frequently used to achieve ventricular rate control in the presence of atrial fibrillation [43]. Large meta-analyses have demonstrated that β-blocker therapy improves survival and reduces hospitalizations in patients with HF who remain in sinus rhythm; however, these benefits appear less evident in individuals with concomitant AF [44]. This discrepancy likely reflects the adverse prognostic impact of AF rather than a lack of therapeutic value of β-blockade itself. Supporting this hypothesis, a propensity-matched analysis from the AF-CHF trial reported an association between β-blocker use and lower mortality among patients with HFrEF and AF, although hospitalization rates were not significantly affected [45]. Consequently, despite some uncertainty regarding their prognostic impact in HF-AF populations, β-blockers remain a key component of HFrEF management and are generally maintained when tolerated due to their established role in neurohormonal modulation and ventricular rate control [43,46].

##### Nondihydropyridine Calcium-Channel Blockers

The use of nondihydropyridine calcium channel blockers is discouraged due to their potential to worsen HF and is therefore considered contraindicated [47].

##### Digoxin

Digoxin may be considered as an alternative or adjunctive option for ventricular rate control in patients with AF and HF, particularly when beta-blockers alone are insufficient or poorly tolerated [48]. Clinical evidence suggests that digoxin can effectively lower heart rate in AF; for example, the DAAF trial demonstrated that intravenous digoxin reduced heart rate by more than 12 beats/min within two hours compared with placebo [49]. In patients with AF and HFrEF, combination therapy with digoxin and beta-blockers has been shown to provide better rate control and symptom improvement than either agent alone [50] (Table 1).

Further evidence from the RATE-AF trial indicated that digoxin achieved similar quality-of-life outcomes compared with bisoprolol, while being associated with fewer adverse events, greater improvement in symptom scores, and lower natriuretic peptide levels [51,52]. Randomized controlled trials have not demonstrated an increase in all-cause mortality with digoxin therapy, although observational studies have reported a possible association between digoxin use and increased mortality [48,53,54,55]. Because of its narrow therapeutic window, careful dosing and monitoring of serum digoxin concentrations—particularly in elderly patients, those with renal dysfunction, or those receiving interacting medications—are recommended to reduce the risk of toxicity [48,56].

##### Amiodarone

Although mainly used for rhythm control, amiodarone may also help achieve ventricular rate control in patients with AF and rapid ventricular response, particularly in critically ill patients with HF where hypotension limits the use of other agents [57]. However, its negative inotropic effects may worsen cardiac function, and conversion to sinus rhythm can occur, requiring careful consideration of thromboembolic risk [58]. Because of its broad extracardiac toxicity and cumulative dose–related adverse effects, amiodarone is generally reserved for cases in which adequate rate control cannot be achieved with other therapies [48].

#### 3.1.2. Rhythm Control

Earlier studies did not show a clear advantage of rhythm over rate control in patients with AF and HFrEF (37). However, with advances in pharmacological therapy and catheter ablation, rhythm control has gained increasing relevance, mainly in patients with suspected arrhythmia-induced cardiomyopathy.

The main approaches to rhythm control in patients with AF and HFrEF include:

##### Antiarrhythmic Drugs

Managing AF in patients with HFrEF is challenging due to the limited safe pharmacologic options and the proarrhythmic or negative inotropic potential of many antiarrhythmic drugs. Although catheter ablation is often preferred in suitable candidates, antiarrhythmic drugs remain an important option for rhythm management, either as standalone therapy, in combination, or as a bridge to more definitive interventions.

Antiarrhythmic Drugs in AF and HFrEF: Recommended Agents

Amiodarone and dofetilide, two class III antiarrhythmic agents, are recommended for patients who require long-term rhythm control therapy [48,59]. These drugs have demonstrated efficacy and an acceptable safety profile in individuals with AF and HFrEF and have also been associated with improvements in LVEF [42,60,61,62].

Amiodarone

Amiodarone has also been extensively studied in HFrEF and is commonly used in patients with AF and HFrEF because it carries a lower risk of proarrhythmia. The CHF-STAT trial demonstrated that patients who converted to sinus rhythm with amiodarone had lower mortality [58]. In the AF-CHF trial, symptomatic HFrEF patients with AF (33% paroxysmal, 67% persistent) were randomized to rhythm versus rate control, with 82% of the rhythm control group receiving amiodarone [63]. There was no difference in all-cause mortality, cardiovascular mortality, or worsening HF. However, AF recurred in 58% of patients in the rhythm control group, while 30% of patients in the rate control group were in sinus rhythm at follow-up, highlighting the limitations of pharmacologic rhythm control [23].

The RACE 3 trial, which included patients with HFrEF and HFpEF with early persistent AF, showed that targeted therapy of underlying conditions plus rhythm control was effective in about half of patients at 1 year [64]. Amiodarone was the most effective drug, maintaining sinus rhythm in 58% of patients, compared with 32% for flecainide and 23% for sotalol or dronedarone, underscoring its relative superiority in HFrEF [23].

Nonetheless, its safety remains controversial, with multiple observational studies and trials reporting increased mortality in those treated with the drug [65,66]. By contrast, recent randomized studies indicate that catheter ablation provides better outcomes than medical therapy in patients with AF and HFrEF [25,67,68,69].

Dofetilide

Dofetilide was evaluated in 1581 symptomatic patients with HFrEF (NYHA class II–IV) in the placebo-controlled DIAMOND-CHF trial to assess safety and potential effects on mortality and morbidity [60]. While dofetilide reduced HF hospitalizations, it had no effect on overall mortality. Among the 25.8% of patients with AF, dofetilide restored sinus rhythm in 12% of subjects within one month, significantly more than placebo (1%) [70].

However, it has not been directly compared with amiodarone, and does not confer a survival benefit. Moreover, its use, as with other agents in this class, is limited by the risk of serious adverse effects and proarrhythmia, which is particularly relevant in the HFrEF population [60].

Antiarrhythmic Drugs in AF and HFrEF: Contraindicated Agents

The CAST trial demonstrated that Class IC antiarrhythmic drugs carry an increased risk of proarrhythmia, leading to their contraindication in patients with ischemic heart disease and reduced LVEF [71].

Other Class III Antiarrhythmics: Sotalol and Dronedarone

Sotalol

Sotalol is a class III antiarrhythmic with additional β-blocking activity, capable of reducing AF recurrence without the extracardiac toxicities typically associated with Amiodarone [72]. However, due to its QT-prolonging effect, treatment initiation requires in-hospital monitoring with electrocardiographic and renal function surveillance [73]. In patients with HFrEF, the role of sotalol is limited. These patients are usually already treated with β-blockers as part of guideline-directed therapy, and the additional β-blockade from sotalol may reduce tolerability, particularly at higher doses required for antiarrhythmic efficacy [74]. Moreover, the risk of proarrhythmia, especially torsades de pointes, is increased in the setting of reduced ejection fraction [74]. Importantly, sotalol has been associated with higher mortality compared with placebo or no treatment, which has led to a weaker recommendation in recent guidelines [72]. Consequently, despite its efficacy and accessibility, its use in HFrEF is generally avoided in favor of safer alternatives.

Dronedarone

Dronedarone is a multichannel class III antiarrhythmic agent developed as a structural analogue of Amiodarone, with the aim of maintaining antiarrhythmic efficacy while reducing extracardiac toxicity. Randomized trials have demonstrated its capacity to maintain sinus rhythm in patients with AF [75,76]. However, its use in HFrEF is constrained by safety concerns. Evidence from the ANDROMEDA trial showed an increase in mortality driven by worsening HF in patients with severe left ventricular systolic dysfunction, leading to contraindication in individuals with advanced HF or recent decompensation [77]. Consequently, current recommendations advise against the use of dronedarone in patients with NYHA class III–IV HF or unstable clinical status, particularly in the presence of reduced ejection fraction [46]. In contrast, data from the ATHENA trial provide a more nuanced perspective; in a subgroup of patients with stable HFrEF (LVEF ≤40%, NYHA II–III), dronedarone did not increase mortality compared with placebo, suggesting that the excess risk is primarily confined to patients with advanced or recently decompensated HF [78]. Nonetheless, given the signal of harm in more severe disease and the availability of safer alternatives, its use in HFrEF remains restricted and should be considered only in carefully selected, clinically stable patients. Overall, while dronedarone retains antiarrhythmic efficacy with a more favorable extracardiac safety profile, its indication in HFrEF is limited by trial evidence demonstrating increased mortality in severe or unstable HF, supporting its contraindication in this population [46].

##### Atrioventricular Nodal Ablation and Pacing

Atrioventricular (AV) node ablation combined with permanent pacing represents a definitive rate-control strategy in patients with AF who remain symptomatic despite optimal pharmacological therapy or in whom such therapy is not tolerated [79]. By eliminating AV conduction, this approach ensures ventricular rate regularization and has consistently been associated with improvements in symptoms, exercise capacity, and quality of life [80].

In patients with HFrEF, the choice of pacing modality is critical. Conventional right ventricular (RV) pacing may worsen ventricular dyssynchrony and negatively impact outcomes; therefore, cardiac resynchronization therapy (CRT) is preferred. CRT has been shown to reduce mortality and HF events compared with RV pacing in patients with reduced ejection fraction [81,82,83]. Accordingly, in patients with HFrEF undergoing AV node ablation and requiring near-complete ventricular pacing, biventricular pacing is recommended to preserve ventricular synchrony and optimize outcomes [82,83,84].

Randomized and observational data support the clinical benefit of this “ablate-and-pace” strategy. In the APAF-CRT trial, the combination of CRT and AV node ablation was superior to pharmacological rate control in reducing mortality and HF hospitalizations, while also improving functional status and symptom burden [85]. Similarly, in patients with pre-existing CRT and suboptimal biventricular pacing due to AF, AV node ablation has been associated with improved symptom control and a significant reduction in all-cause mortality [86,87,88]. Overall, compared with medical rate control alone, this strategy provides a more consistent ventricular response and improved clinical outcomes in appropriately selected patients.

Emerging pacing strategies targeting the conduction system, such as His bundle pacing or left bundle branch area pacing, may offer physiological activation and potentially comparable or superior resynchronization compared with conventional CRT, although current evidence remains limited and largely observational [83,89,90].

Despite these benefits, several limitations must be considered. AV node ablation is irreversible and results in lifelong pacemaker dependency, with potential risks related to device malfunction, lead complications, or infection [91]. In addition, this approach does not restore sinus rhythm, and some patients may continue to experience AF-related symptoms. Notably, in the PABA-CHF trial, catheter ablation (pulmonary vein isolation) was superior to the ablate-and-pace strategy in improving left ventricular ejection fraction, highlighting that rhythm control may be preferable in selected patients [92].

In summary, AV node ablation combined with CRT is an effective and well-supported strategy for rate control in patients with AF and HFrEF when pharmacological therapy fails, offering significant improvements in symptoms and clinical outcomes, provided that appropriate pacing techniques are employed.

##### AF Ablation

Catheter ablation has become an increasingly used strategy for rhythm control in patients with AF and HFrEF, supported by a growing evidences from randomized data [93,94]. Several trials have compared ablation with rate control strategies and have shown improvements in cardiac function and clinical status. In the PABA-CHF trial, pulmonary vein isolation (PVI) was compared with atrioventricular node ablation combined with biventricular pacing in patients with drug-refractory AF and HFrEF, demonstrating a higher LVEF (35% vs. 28%, *p* < 0.001) and better symptom control in the ablation group [83]. Similarly, the CAMERA-MRI trial evaluated catheter ablation versus medical rate control in patients with AF and systolic dysfunction without an alternative cause, reporting a substantial increase in LVEF in the ablation arm (32% to 50%) compared with more modest changes under rate control (34% to 39%, *p* < 0.0001) [25]. These findings suggest that, in selected patients, a component of ventricular dysfunction may be reversible after restoration of sinus rhythm [25].

Trials comparing catheter ablation with antiarrhythmic drug therapy have also shown favorable results, although with some variability. In the AATAC trial, patients with persistent AF and HFrEF were randomized to catheter ablation or amiodarone; ablation was associated with higher rates of sinus rhythm maintenance and lower rates of hospitalization and all-cause mortality [69]. However, these findings should be interpreted in the context of the study population and design, including selection criteria and follow-up duration.

The CASTLE-AF trial provided further evidence by comparing catheter ablation with conventional medical therapy (rate or rhythm control) in patients with symptomatic AF and LVEF ≤ 35%. Ablation was associated with a reduction in the composite endpoint of all-cause mortality and HF hospitalization (HR: 0.62; *p* = 0.007), as well as improvements in LVEF and functional capacity [68]. Notably, this was one of the first trials designed to evaluate hard clinical outcomes, although the relatively selected study population may limit generalizability.

More recent data have extended these observations to patients with advanced HF. In the CASTLE-HTx trial, catheter ablation was compared with guideline-directed medical therapy in patients eligible for transplantation or mechanical support, showing a lower incidence of the composite endpoint of death, urgent transplantation, or left ventricular assist device implantation in the ablation group [95].

At the same time, not all studies have demonstrated consistent benefit. The AMICA trial did not show a significant improvement in LVEF with catheter ablation compared with medical therapy in patients with persistent AF and advanced systolic dysfunction [96,97]. In the broader AF population, the CABANA trial did not demonstrate a significant reduction in the primary composite endpoint in the intention-to-treat analysis, although secondary analyses suggested potential benefit in selected subgroups, including patients with HF [97,98]. These discrepancies highlight the importance of patient selection and study design when interpreting results.

Meta-analyses of randomized trials generally support an association between catheter ablation and improvements in LVEF, as well as reductions in hospitalizations and, in some analyses, mortality [99,100,101]. However, the magnitude of benefit varies across studies, and residual uncertainty remains regarding the extent to which these effects translate into consistent prognostic advantage in all patient subgroups.

The timing of intervention may also influence outcomes. The EAST-AFNET 4 trial showed that early rhythm control, initiated within the first year after AF diagnosis, was associated with a reduction in cardiovascular events, although catheter ablation was used in a minority of patients [102]. Observational data suggest that earlier ablation may be associated with better rhythm control and improvements in ventricular function, particularly in patients with reduced baseline LVEF [103,104,105]. Nevertheless, the optimal timing of ablation in HFrEF remains to be clearly defined.

Overall, available evidence suggests that catheter ablation can improve symptoms, ventricular function, and, in selected populations, clinical outcomes in patients with AF and HFrEF. However, results are not uniform across all trials, and careful patient selection remains essential when considering this approach [48,59].

### 3.2. Stroke Prevention

HF is recognized as a significant contributor to thromboembolic risk in patients with AF and is incorporated into widely used risk stratification tools such as the CHA_2_DS_2_-VA score [48]. In the presence of HFrEF, this risk is further amplified, with estimates suggesting a 1.7- to 2.6-fold increase [106]. In a large Medicare-based cohort including patients with newly diagnosed AF, a total of 47,840 patients had HFrEF, compared with 32,360 with HFpEF and 718,392 without HF. Patients with HFrEF exhibited a higher burden of comorbidities and significantly elevated thromboembolic and bleeding risk profiles, reflected by higher CHA_2_DS_2_-VASc and HAS-BLED scores. Even after multivariable adjustment for baseline characteristics and oral anticoagulation (OAC) therapy, HFrEF remained independently associated with a significantly increased risk of ischemic stroke, myocardial infarction, and heart failure hospitalization compared with patients without HF [107]. Moreover, patients with HFrEF demonstrated a higher risk of all-cause mortality and HF hospitalization compared with those with HFpEF. These findings highlight the substantial prognostic burden of HFrEF in AF populations and underscore the persistent risk of adverse cardiovascular events despite anticoagulation therapy [107].

Current guidelines recommend oral anticoagulation in patients with HF and concomitant AF [41,48]. Although the combination of HF and AF is associated with increased stroke severity and higher all-cause mortality, available evidence suggests that the overall risk of stroke does not significantly differ among HF subtypes [41,108].

Left atrial appendage occlusion (LAAO) may be considered in patients with AF who have a high thromboembolic risk but contraindications to OAC. In national registries, approximately 40% of patients undergoing percutaneous LAAO have HF, a higher proportion than in clinical trials (~25%) [109,110]. However, HF has been associated with higher rates of procedural complications and increased long-term mortality, making the overall benefit of percutaneous LAAO in this population uncertain [111,112]. Surgical LAAO, often performed during concomitant cardiac surgery, has been shown to reduce stroke risk when combined with postoperative OAC [113]. Although concerns exist that LAA removal could worsen HF by reducing atrial natriuretic peptide release, clinical evidence has not confirmed this effect [114].

## 4. Management Challenges of Guideline-Directed Therapy in Patients with HFrEF and Atrial Fibrillation

Guideline-directed medical therapy (GDMT) for HFrEF is structured around four major therapeutic pillars: renin–angiotensin system inhibition (ACE inhibitors, angiotensin receptor blockers, or preferably angiotensin receptor–neprilysin inhibitors), beta-blockers, mineralocorticoid receptor antagonists, and sodium–glucose cotransporter-2 inhibitors. These therapies have consistently demonstrated reductions in mortality and HF hospitalizations and are recommended irrespective of the presence of HF. However, their interaction with AF is complex [115].

### 4.1. Renin–Angiotensin System Inhibition and Angiotensin Receptor–Neprilysin Inhibitors

Renin–angiotensin system inhibition represents a core component of guideline-directed medical therapy in HFrEF, with established benefits on mortality and HF hospitalizations [116,117,118,119,120]. Beyond their hemodynamic effects, these therapies may also influence the development and progression of AF through modulation of structural remodeling, fibrosis, and atrial pressure [121,122,123,124].

#### 4.1.1. ACE Inhibitors and Angiotensin Receptor Blockers (ARBs)

Evidence supporting a role in AF prevention with ACE inhibitors and ARBs comes largely from secondary analyses of randomized trials. In the SOLVD study, enalapril reduced the incidence of new-onset AF compared with placebo (5.4% vs. 24%) [125]. Similarly, trandolapril was associated with a lower risk of AF in patients with left ventricular systolic dysfunction following myocardial infarction in the TRACE trial (2.8% vs. 5.3%) [126]. Among ARBs, valsartan reduced AF occurrence in Val-HeFT (5.1% vs. 8.0%; HR: 0.63), while candesartan in the CHARM program showed a modest reduction in incident AF across a broad HF population [127,128].

Despite these findings, the evidence is derived primarily from post hoc analyses rather than trials specifically designed to assess AF outcomes, and the magnitude of benefit varies. Moreover, while RAAS inhibition appears to reduce incident AF, its impact on outcomes in patients with established AF is less well defined [129].

#### 4.1.2. Angiotensin Receptor–Neprilysin Inhibitors (ARNI)

Sacubitril/valsartan has demonstrated superiority over ACE inhibitors in reducing mortality and HF hospitalizations in HFrEF, but its effects on AF are less consistent [130,131]. Mechanistically, neprilysin inhibition may influence atrial remodeling and calcium handling, potentially affecting arrhythmogenesis [132,133,134].

However, large clinical trials have not demonstrated a clear reduction in AF incidence. In PARADIGM-HF, the occurrence of new-onset AF was similar between sacubitril/valsartan and enalapril [135,136].

Some smaller studies and observational data suggest a potential role in reverse atrial remodeling, including reductions in left atrial size and improvements in atrial function, particularly in patients undergoing catheter ablation [137,138,139]. Importantly, the prognostic benefits of sacubitril/valsartan in HFrEF appear to be maintained regardless of AF status [131,136].

Overall, while both RAAS inhibitors and ARNI play a central role in HFrEF management, their effects on AF differ: ACE inhibitors and ARBs have more consistent evidence for prevention of incident AF, whereas ARNI therapy has not demonstrated a clear reduction in AF incidence, despite its superior impact on HF outcomes.

### 4.2. Beta-Blockers

Beta-blockers are a cornerstone of HFrEF therapy and are also first-line agents for rate control in AF [47]. Mechanistically, they counteract sympathetic overactivation, reduce atrial pressure, and may limit electrical and structural remodeling, thereby potentially reducing AF susceptibility [140].

Evidence suggests that beta-blockers may reduce the incidence of AF in patients with HFrEF. In the CAPRICORN trial, carvedilol was associated with a lower incidence of AF and atrial flutter (HR: 0.48) [141]. Similarly, meta-analyses of randomized trials have shown a reduction in new-onset AF with beta-blocker therapy [44,142].

However, their impact on prognosis in patients with established AF is less clear. Patient-level meta-analyses have shown that while beta-blockers reduce mortality in patients with HFrEF in sinus rhythm, this benefit was not observed in those with AF (HR: 0.97; *p* = 0.73) [44]. These findings suggest that the prognostic effect of beta-blockers may depend on underlying rhythm.

In contrast, observational studies and secondary analyses have reported an association between beta-blocker use and reduced mortality in patients with AF and HF, although these results may be influenced by residual confounding and differences in patient characteristics [45,143].

Taken together, beta-blockers remain essential for rate control and symptomatic management in AF, but their impact on long-term outcomes in patients with AF and HFrEF remains uncertain and may vary depending on patient selection and underlying rhythm.

### 4.3. Mineralocorticoid Receptor Antagonists (MRAs)

Mineralocorticoid receptor activation contributes to atrial fibrosis and electrical remodeling, providing a mechanistic link between aldosterone and AF development. Experimental studies have shown that aldosterone promotes atrial fibrosis and conduction abnormalities, while MRAs may counteract these effects [115].

Clinical data suggest that MRAs may reduce AF incidence, although results are heterogeneous. In EMPHASIS-HF, eplerenone reduced the occurrence of new-onset AF compared with placebo [115,144]. Meta-analyses have also reported lower AF rates with MRA therapy, although with significant heterogeneity across studies [145].

In contrast, no reduction in AF incidence was observed with spironolactone in the TOPCAT trial, highlighting potential differences between agents and patient populations [115,146]. More recently, finerenone has shown a reduction in incident AF in patients with chronic kidney disease and diabetes, suggesting possible class effects with newer agents [147].

Overall, MRAs may have a role in modulating atrial remodeling and reducing AF risk, but evidence remains inconsistent and may depend on the specific agent and patient population.

### 4.4. SGLT2 Inhibitors

SGLT2 inhibitors improve outcomes across the spectrum of HF, but their effects on AF remain less well defined [52]. Proposed mechanisms include volume reduction, modulation of autonomic balance, improved myocardial energetics, and reduction in atrial remodeling [148,149].

Clinical data are inconsistent. In the DECLARE-TIMI 58 trial, dapagliflozin reduced the incidence of AF and atrial flutter, whereas in DAPA-HF no reduction in incident AF was observed despite clear benefits on HF outcomes [149]. Similar neutral findings regarding AF incidence were reported in EMPEROR-Preserved and DELIVER, although treatment benefits on heart failure outcomes were consistent regardless of AF status [150].

Observational data suggest potential benefits in reducing AF burden and recurrence, including after catheter ablation, but these findings require confirmation in dedicated randomized studies [150].

Thus, while SGLT2 inhibitors are a key component of GDMT in HFrEF, their role in AF prevention or management remains uncertain.

## 5. Prognosis of Patients with Coexisting Atrial Fibrillation and HFrEF

The coexistence of AF and HFrEF has been associated with an increased risk of mortality and hospitalization compared with either condition alone, although the relative contribution of each condition to these adverse outcomes remains difficult to establish. Data from the Framingham Heart Study showed an increased risk of death in patients with both diagnoses (adjusted HR ≈ 3.6), with a median survival of approximately 3 years. Early mortality is high, with nearly one-third of patients dying within the first year, and long-term mortality exceeding 60% at 5 years. Mortality is highest when AF and HF are diagnosed concurrently, suggesting more advanced disease [151].

In patients with AF, reduced ejection fraction further worsens outcomes. In the ENGAGE-AF TIMI 48 trial, patients with HFrEF had higher rates of HF hospitalization and mortality compared with those with preserved EF [152]. In addition, AF burden is an important determinant of prognosis, as increasing AF burden is associated with higher rates of hospitalization and death. Progression from subclinical to sustained AF is also linked to worse outcomes [153,154].

Data from the RIF-CHF registry confirm the high-risk profile of this population. Patients with AF and HFrEF had the highest cardiovascular mortality (15.5%) and adverse composite outcomes (25.5%) at 1 year [155]. In this group, prognosis is mainly related to HF severity, comorbidities, and structural heart disease rather than AF alone [155,156].

Predictors of HF hospitalization include diabetes, hypertension, vascular disease, prior stroke, coronary artery disease, signs of congestion, and longer duration of AF and HF, as well as persistent AF and higher CHA_2_DS_2_-VASc and HAS-BLED scores [155]. Predictors of cardiovascular mortality include anemia, renal dysfunction, prior myocardial infarction or stroke, right atrial enlargement, and echocardiographic evidence of myocardial damage [155]. Predictors of ischemic events include older age, vascular disease, hypertension-related changes, abnormal liver function, and prior ischemic events [155].

The sequence of disease onset also influences prognosis. HF preceding AF is associated with worse outcomes, suggesting that AF may reflect more advanced myocardial disease in this setting [157].

Overall, in patients with AF and HFrEF, prognosis is primarily determined by HF severity and associated comorbidities, while AF acts as an additional risk factor with variable independent impact.

## 6. Conclusions

This narrative review highlights the complex interplay between AF and HFrEF. Their coexistence is associated with increased morbidity and mortality, although the relative contributions of HF severity, AF burden, and associated comorbidities to these outcomes remain difficult to disentangle. Management remains challenging and should be individualized according to clinical presentation and the likely contribution of AF to ventricular dysfunction. Rate control remains an important therapeutic strategy, whereas rhythm control, particularly catheter ablation, has been associated with improvements in left ventricular function and clinical outcomes in selected populations. However, the magnitude of benefit varies across studies and patient populations, while pharmacological rhythm control remains constrained by safety and efficacy considerations. Guideline-directed medical therapy remains the cornerstone of HFrEF treatment; however, the effects of individual therapeutic classes on AF occurrence and AF-related outcomes are heterogeneous.

Overall, current evidence supports a heterogeneous interaction between AF and HFrEF rather than a uniformly reciprocal relationship in all patients. AF may predominantly contribute to ventricular dysfunction in some patients, whereas in others it may primarily reflect an advanced underlying HF substrate; between these extremes, both conditions may reinforce each other through a self-perpetuating cycle. Further studies are needed to identify the dominant mechanisms in individual patients, refine patient selection and timing for rhythm-control strategies, and determine whether a more phenotype-oriented approach may improve long-term outcomes.

## Figures and Tables

**Table 1:** Catheter Ablation Versus Medical and Device-Based Therapies in AF With HFrEF: Evidence From Randomized Trials.

Trial (Year)	N	Population	Comparator	Main Endpoint	Conclusions
PABA-CHF (2008)	81	AF, LVEF ≤ 40%, NYHA II–III	NYHA II–III AV node ablation + CRT	Functional composite (LVEF, 6MWD, QoL)	Ablation superior for LVEF, exercise capacity, and QoL; high sinus rhythm maintenance
MacDonald (2011)	41	Persistent AF, LVEF < 35%	Rate control	LVEF	No significant LVEF improvement; signal only in secondary analyses; notable complications
ARC-HF/Jones (2013)	52	Persistent AF, LVEF ≤ 35%	Rate control	Peak VO_2_	Moderate improvement in exercise capacity and QoL with ablation
CAMTAF/Hunter (2014)	50	Persistent AF, LVEF < 50%	Rate control	LVEF	Significant improvement in LVEF and functional status
AATAC (2016)	203	Persistent AF, LVEF ≤ 40%, ICD/CRT-D	Amiodarone	AF recurrence	Superior rhythm control vs. amiodarone; reduced mortality and hospitalizations
CAMERA-MRI (2017)	66–68	Persistent AF + idiopathic cardiomyopathy	Rate control	LVEF (CMR)	Marked LVEF recovery; greater benefit without myocardial fibrosis
CASTLE-AF (2018)	303–363	AF, LVEF ≤ 35%, ICD/CRT-D	Medical therapy (rate/rhythm)	Death + HF hospitalization	Significant reduction in mortality and HF admissions
AMICA (2019)	140	Persistent AF, LVEF ≤ 35%	Medical therapy	LVEF	No significant difference between strategies
RAFT-AF (2022)	411	AF + HF (elevated NT-proBNP)	Rate control	Death + HF events	Non-significant trend toward benefit; improved LVEF and functional status
CASTLE-HTx (2023)	194	AF, end-stage HF	Medical therapy	Death/LVAD/transplant	Marked reduction in major adverse outcomes with ablation
CRABL-HF (2024)	110	AF, LVEF ≤ 40%	Cryo vs. RF ablation	AF recurrence	Cryoablation non-inferior to RF ablation

## Data Availability

The raw data supporting the conclusions of this article will be made available by the authors on request.
